# Increased number of negative lymph nodes is associated with improved cancer specific survival in pathological IIIB and IIIC rectal cancer treated with preoperative radiotherapy

**DOI:** 10.18632/oncotarget.2560

**Published:** 2014-10-20

**Authors:** Qingguo Li, Changhua Zhuo, Guoxiang Cai, Dawei Li, Lei Liang, Sanjun Cai

**Affiliations:** ^1^ Department of Colorectal Surgery, Fudan University Shanghai Cancer Center, Shanghai, People's Republic of China; ^2^ Department of Oncology, Shanghai Medical College, Fudan University, Shanghai, People's Republic of China; ^3^ Department of Surgical Oncology, Fujian Provincial Cancer Hospital, Teaching Hospital of Fujian Medical University, Fuzhou, People's Republic of China

**Keywords:** Rectal cancer, negative lymph nodes, prognosis, SEER

## Abstract

Preoperative radiation significantly decreases the number of retrieved lymph nodes (LNs) in rectal cancer, but little is known with respect to the prognostic significance of negative LN (NLN) counts under these circumstances. In this study, Surveillance, Epidemiology, and End Results Program (SEER)-registered ypIII stage rectal cancer patients, and patients from Fudan University Shanghai Cancer Center (FDSCC) were combined and analyzed. The results showed that the survival rate of patients with *n* (cutoff) or more NLNs increased gradually when *n* ranged from two to nine. After *n* reached 10 or greater, survival rates were approximately equivalent. Furthermore, the optimal cutoff value of 10 was validated as an independent prognostic factor in stage ypIIIB and ypIIIC patients by both univariate and multivariate analysis (*P* < 0.001); the number of NLNs could also stratify the prognosis of ypN(+) patients in more detail. Patients in the FDSCC set validated these findings and confirmed that NLN count was not decreased in the good tumor regression group relative to the poor tumor regression group. These results suggest that NLN count is an independent prognostic factor for ypIIIB and ypIIIC rectal cancer patients, and, together with the number of positive LNs, this will provide better prognostic information than the number of positive LNs alone.

## INTRODUCTION

Preoperative radiation (preop-RT) following curative resection has become a standard method to treat locally advanced rectal cancer because of lowered local recurrence rates [1, 2]. Several studies have reported a decrease in the number of lymph nodes (LNs) retrieved, as well as fewer lymph node metastases, after preop-RT [3–6]. Moreover, a decreased number of LNs is related to good tumor response, and thus improved cause specific survival (CSS) [7–10] and survival are not influenced by the number of LNs obtained. Further, patients with less than 12 LNs retrieved might have more favorable disease free survival rate than those with 12 or more LNs assessed [7, 11], but some studies continue to support the theory that identifying more LNs results in better survival [12, 13]. The total number of LNs (TLN) retrieved comprises both positive and negative LNs (NLNs), so the relationship between TLNs and prognosis is confounded by the prognostic effect of the number of positive LNs (PLNs). The concept of NLN counts has recently attracted attention as a prognostic indicator in colon [14], gastric [15], esophageal [16], and cervical [17]. However, there is no research focusing on the correlation between NLN counts and patient prognosis in the setting of rectal cancer treated with preop-RT.

Given the growing importance of preop-RT in the management of patients with rectal cancer, we designed our study to specifically assess the effect of the number of NLNs on CSS in patients with stage ypIII rectal cancer by analyzing the Surveillance, Epidemiology, and End Results (SEER)-registered database. Moreover, because SEER data lacks information on neoadjuvant chemoradiation therapy (NCRT) methods, NCRT response, and quality of surgery, we further clarified these relevant issues in another set of patients with locally advanced rectal cancer from the Fudan University Shanghai Cancer Center (FDSCC).

## RESULTS

### SEER database patient characteristics

A total of 1,712 eligible patients during the 8-year study period were indentified, including 1086 male and 626 female patients. There were 223 patients (13%) with ypIIIA stage, 1044 patients (61%) with ypIIIB stage, and 445 patients (26%) with ypIIIC stage rectal cancer. Patient demographics and pathological features are summarized in Table 1. The proportion of high/moderate differentiation gradually decreased from ypIIIA to ypIIIC (74.9% to 59.6%), and the same phenomena was found with respect to histotype; the percentage of adenocarcinoma decreased from 90.6% in ypIIIA to 69.0% in ypIIIC.

**Table 1:** Demographic and tumor characteristics of patients with stage ypIII rectal cancer

			AJCC Subgroup					
	All Patients		ypIIIA		ypIIIB		ypIIIC	
	n=1712		n=223		n=1044		n=445	
Characteristic	No.	%	No.	%	No.	%	No.	%
**Sex**								
Male	1086	63.4%	142	63.7%	676	64.8%	268	60.2%
Female	626	36.6%	81	36.3%	368	35.2%	177	39.8%
**Age**								
<40	139	8.1%	6	2.7%	86	8.2%	47	10.6%
≥40	1573	91.9%	217	97.3%	958	91.8%	398	89.4%
**Race**								
Caucasian	1393	81.4%	191	85.7%	852	81.6%	350	78.7%
Non- Caucasian	319	18.6%	32	14.3%	192	18.4%	95	21.3%
**Pathological grading**								
High/Moderate	1176	68.7%	167	74.9%	744	71.3%	265	59.6%
Poor/Anaplastic	395	23.1%	41	18.4%	218	20.9%	136	30.6%
Unknown	141	8.2%	15	6.7%	82	7.9%	44	9.9%
**Histotype**								
Adenocarcinoma	1377	80.4%	202	90.6%	868	83.1%	307	69.0%
Mucinous /Signet ring cell	335	19.6%	21	9.4%	176	16.9%	138	31.0%
**ypT stage**								
T1/T2	236	13.8%	223	100%	13	1.2%	/	/
T3/4	1476	86.2%	/	/	1031	98.8%	445	100%
No. of LNs dissected	11.6		10.6		10.7		14.2	
No. of positive LNs	3.5		2.0		2.2		7.4	
No. of negative LNs	8.1		8.6		8.5		6.8	

The median number of NLNs for the cohort was 11.6 and the median number of PLNs was 3.5. As expected, patients with stage ypIIIIC cancer had more PLNs (median of 7.4) than patients with stage ypIIIB (median of 2.2) or stage ypIIIA cancer (median of 2.0). Conversely, there were fewer NLNs in patients with stage ypIIIC cancer (median of 6.8) than those with stage ypIIIB (median of 8.5) or stage ypIIIIA (median of 8.6) cancer, and there was a significant correlation between the number of TLNs and NLNs (r = 0.887, *P* < 0.001). Correlations between the number of NLNs and PLNs were weak or negligible (*r* = −0.097) [18].

### Identification of cutoff points of minimum number of NLNs retrieved in the SEER database

We first treated NLN count as a continuous variable, and it was validated as a significant prognostic factor by univariate log-rank test (*χ*^2^ = 41.05, *P* < 0.001). Next, to assess the influence of different cutoff points on rectal cancer-CSS(RCSS) in ypIII patients, we further analyzed individual NLN counts from 2 to 18. The 5-year RCSS of patients with *n* (cutoff point) or more nodes and less than *n* nodes were calculated, respectively. The survival rate of patients with *n* or more nodes increased gradually when *n* ranged from two to nine and the difference in survival was most significant (maximum of χ^2^ log-rank values) for five nodes. Patients with five or more NLNs evaluated had a relative reduction of 17.2% for death from rectal cancer than those with five or fewer NLNs evaluated (66.9% versus 49.7%, χ^2^ = 58.114, *P* < 0.0001). After 10 NLNs, the survival rates were roughly equal (Table 2). It is likely that 10 is the optimal cutoff value, at and above which the influence of NLNs count on survival is minimal. There was an absolute 25.0% improvement in 5-year RCSS if ≥10 NLNs were analyzed compared with those who had <2.

**Table 2:** Univariate analysis for the influence of different cutoffs on RCSS in stage ypIII rectal cancer

Cutoff	No.	5-year RCCS	Log-rank χ^2^	*P* value
<2	251	45.1%	40.604	<0.001
≥2	1461	63.1%		
<3	377	47.4%	41.780	<0.001
≥3	1335	64.2%		
<4	506	49.3%	47.383	<0.001
≥4	1206	65.2%		
<5	643	49.7%	58.114	<0.001
≥5	1069	66.9%		
<6	775	52.1%	50.196	<0.001
≥6	937	67.4%		
<7	889	53.6%	48.125	<0.001
≥7	823	68.0%		
<8	988	54.6%	68.6%	<0.001
≥8	724	68.6%		
<9	1076	55.4%	40.856	<0.001
≥9	636	69.0%		
<10	1164	56.0%	37.411	<0.001
≥10	548	70.1%		
<11	1241	56.3%	31.093	<0.001
≥11	471	70.2%		
<12	1305	57.7%	25.762	<0.001
≥12	407	69.7%		
<13	1356	57.8%	26.407	<0.001
≥13	356	70.8%		
<14	1401	58.5%	17.277	<0.001
≥14	311	69.4%		
<15	1454	58.7%	19.285	<0.001
≥15	258	70.7%		
<16	1488	59.1%	14.224	<0.001
≥16	224	69.8%		
<17	1522	59.2%	13.351	<0.001
≥17	190	70.9%		
<18	1548	59.5%	10.450	0.001
≥18	164	70.6%		

### Effect of the number of NLNs on RCSS in the SEER database

The number of NLNs and other clinicopathological factors, including early year of diagnosis *(P* = 0.001), poor and undifferentiated tumor grade (*P* = 0.002), mucinous and signet-ring cancer (*P* < 0.001), and number of PLNs (*P* < 0.001), were significant risk factors for poor survival on univariate analysis (Table 3). Multivariate analysis with Cox regression was performed and year of diagnosis, tumor grade, and PLN and NLN counts (with an optimal cutoff of 10) were independent prognostic factors for RCSS (Table 3), and a higher number of NLNs were found to have a positive effect on survival (hazard ratio [HR] 0.644; 95% confidence interval [CI] 0.549–0.755; Table 3).

**Table 3:** Univariate and multivariate survival analyses for evaluating the influence of the number of NLNs retrieved on RCSS in stage ypIII rectal cancer

		Univariate analysis		Multivariate analysis	
Variable	5-year RCCS	Log rank χ^2^ test	*P*	HR(95%CI)	*P*
**Years of diagnosis**		11.153	0.001		0.008
1988–2001	54.3%			Reference	
2002–2005	63.7%			0.823(0.713–0.951)	
**Sex**		0.053	0.818		NI
Male	60.7%				
Female	60.2%				
**Age**		1.483	0.223		NI
<40	61.3%				
≥40	60.5%				
**Race**		3.707	0.054		NI
Caucasian	61.5%				
Non- Caucasian	56.4%				
**Grade**		9.729	0.002		<0.001
High/Moderate	64.9%			Reference	
Poor/Anaplastic	44.8%			1.574(1.347–1.838)	
Unknown	67.9%			0.845(0.639–1.116)	
**Histotype**		15.830	<0.001		0.201
Adenocarcinoma	62.4%			Reference	
Mucinous/signet ring cell	52.6%			1.117(0.943–1.325)	
**ypT Stage**		64.721	<0.001		<0.001
T1–2	76.2%			Reference	
T3–4a	60.3%			1.544(1.224–1.949)	
T4b	34.7%			2.922(2.163–3.947)	
**No. of NLNs**		37.411	<0.001		<0.001
<10	56.0%			Reference	
≥10	70.1%			0.644(0.549–0.755)	
**No. of PLNs**		86.505	<0.001		<0.001
ypN1	67.4%			Reference	
ypN2	46.3%			1.696(1.470–1.957)	

NI: not included in the multivariate survival analysis.

### Subgroup analysis for evaluating the effect of NLN counts according to yp-TNM and yp-N classification in the SEER database

**Figure 1:**
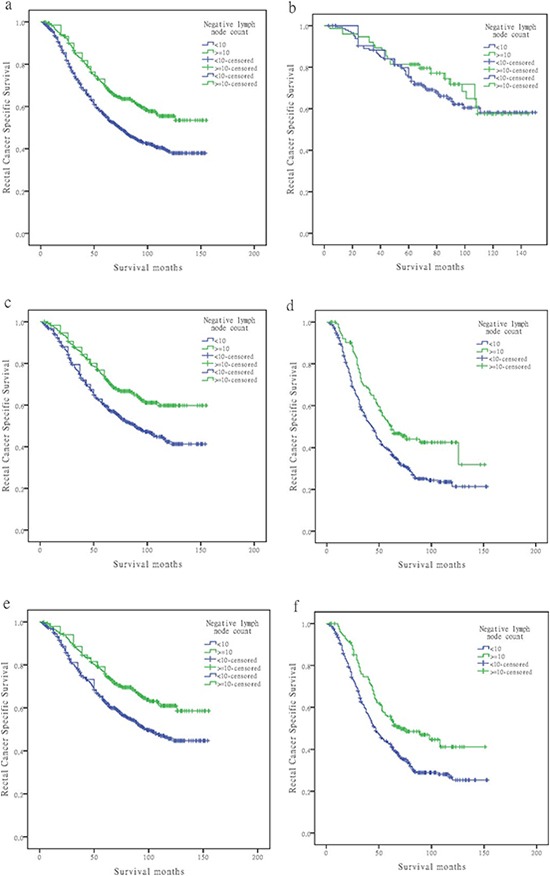
Log-rank tests of cause specific comparing those who had ≥10 negative lymph nodes with those who had <10 negative nodes for **(A)** stage ypIII: 56.0% versus 70.1%, respectively; χ^2^ = 37.411, *P* < 0.001; **(B)** stage ypIIIA: 75.5% versus 81.3%, respectively; χ^2^ = 0.678, *P* = 0.410; **(C)** stage ypIIIB: 60.0% versus 73.9%, respectively; χ^2^ = 21.020, *P* < 0.001; **(D)** stage ypIIIC: 38.4% versus 50.4%, respectively; χ^2^ = 12.905, *P* < 0.001; **(E)** ypN1 stage: 62.9% versus 75.9%, respectively; χ^2^ = 20.659, *P* < 0.001; and **(F)** ypN2 stage: 42.5% versus 55.6%, respectively; χ^2^ = 12.905, *P* = 0.002.

After stratifying patients by yp-TNM stage, for patients with stage ypIIIB and ypIIIC cancers, the difference between <10 and ≥10 NLN counts was statistically significant on both univariate and multivariate analysis (*P* < 0.05); those with 10 or more NLNs retrieved had <70% the mortality rate from rectal cancer than those with 10 or fewer NLNs evaluated (stage IIIB, HR 0.668; 95%CI, 0.541–0.825; *P* < 0.0001; stage IIIC, HR 0.639; 95%CI, 0.480–0.849; *P* = 0.002) regardless of the number of PLNs present. For patients with stage IIIA disease, although there was 5.8% decreased in mortality from rectal cancer in patients with 10 or more NLNs retrieved than those with 10 or fewer NLNs evaluated, the difference wasn't statistically significant (*P* = 0.410; Table 4, Figure 1).

**Table 4:** Univariate and multivariate analyses of NLN count on RCSS based on different cancer stage

		Univariate analysis		Multivariate analysis	
Variable	5-year RCCS	Log rank χ^2^ test	*P*	HR(95%CI)	*P*
**TNM Stage**					
**Stage ypIIIA**					NI
No. of NLNs		0.678	0.410		
<10	75.5%				
≥10	81.3%				
**Stage ypIIIB**					
No. of NLNs		21.020	<0.001		<0.001
<10	60.0%			Reference	
≥10	73.9%			0.668(0.541–0.825)	
**Stage ypIIIC**					
No. of NLNs		11.033	0.001		0.002
<10	38.4%			Reference	
≥10	50.4%			0.639(0.480–0.849)	
**ypN Stage**					
No. of NLNs					<0.001
**N1**		20.659	<0.001		
<10	62.9%			Reference	
≥10	75.9%			0.648(0.528–0.797)	
No. of NLNs					
**N2**		12.905	<0.001		0.002
<10	42.5%			Reference	
≥10	55.6%			0.667(0.517–0.860)	

*P*-values refer to comparisons between two groups and were adjusted for years of diagnosis, sex, age, race, pathological grading, tumor histotype, and the number of PLNs (only in the TNM subgroup analysis) as covariates.

NI: not included in the multivariate survival analysis.

The number of PLNs were significantly associated with RCSS, and further analysis showed that the number of NLNs was an independently prognosis factor for each ypN stage on both univariate and multivariate analysis (*P* < 0.05). For rectal cancer patients with ypN1 stage, there was an absolute 13% improvement in 5-year RCSS if ≥10 NLNs were analyzed compared with to those who had <10 (*P* < 0.001). In patients with ypN2 stage cancer, 5-year RCSS for ≥10 and <10 LNs was 55.6% and 42.5%, respectively (*P* = 0.002; Table 4, Figure 1).

### Evaluating the SEER database outcomes using the FDSCC set

The above results should be treated with caution as they might be biased by confounding factors, such as NCRT response and quality of surgery (palliative resection or radical resection). To evaluate the reliability of SEER results, we studied relevant issues in 108 eligible patients from the FDSCC. Patient demographics and pathological features are summarized in Table 5.

**Table 5:** Demographic and clinical features of patients with rectal cancer (ypIII stage) from Fudan University Shanghai Cancer Center

	n	%
**Age**	52 (23–78)	
**Sex**		
male	76	70.4%
female	32	29.6%
**Histotype**		
Adenocarcinoma	92	85.2%
Mucinous /Signet ring cell	16	14.8%
**ypN stage**		
N1	69	63.9%
N2	39	36.1%
**LNs retrieval**		
<12	64	59.3%
≥12	44	40.7%
**NLNs retrieval**		
<10	77	71.3%
≥10	31	28.7%
**Lymphovascular invasion**		
Negative	82	75.9%
Positive	26	24.1%
**Perineural invasion**		
Negative	79	73.1%
Positive	29	26.9%
**TRG**		
0	24	22.2%
1	32	29.6%
2	30	27.8%
3	12	11.1%
4	10	9.3%

**Figure 2:**
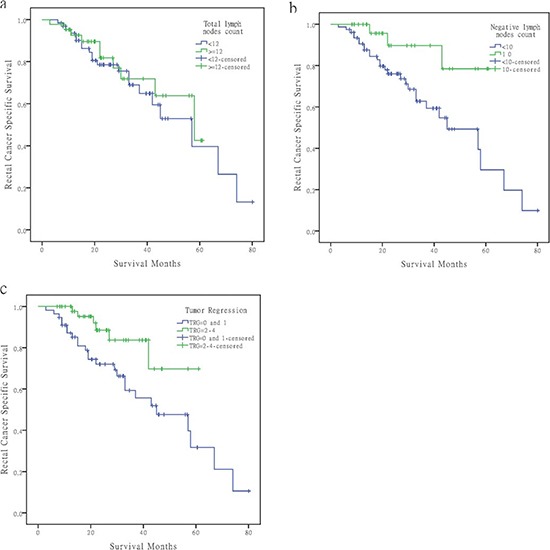
Survival curves of rectal cancer patients at ypIII stage in Fudan University Shanghai Cancer Center according to different factors **(A)** LN count <12 versus ≥12, χ^2^ = 0.302, *P* = 0.582. **(B)** Using 10 as the NLN cutoff, χ^2^ = 5.437, *P* = 0.020. **(C)** Poor regression (TRG0–1) versus good regression (TRG2–4), χ^2^ = 5.911, *P* = 0.0156

First, we studied the 12 TLN cutoff in FDSCC; 3-year RCSS in patients with less than and more than 12 LNs was 68.9% and 71.8%, respectively, which was not statistically significant (χ^2^ = 0.302, *P* = 0.582; Figure 2).

Second, we tested the 10 NLN cutoff in FDSCC; 3-year RCSS in patients with <10 NLNs and ≥10 NLNs was 62.8% and 89.7%, respectively (χ^2^ = 5.437, *P* = 0.020; Figure 2).

Third, we evaluated the correlation between tumor regression grade (TRG) and RCSS; TRG 4, 3, 2, 1, and 0 was found in 10 (9.3%), 12 (11.1%), 30 (27.8%), 32 (29.6%), and 24 (22.2%) of the resected specimens, respectively. The 3-year RCSS was 59.4% in the poor tumor regression group (TRG0–1), which is lower than in the good tumor regression group (TRG2–4; 83.7%), which was statistically significant (χ^2^ = 5.911, *P* = 0.015; Figure 2).

Finally, we investigated the clinical relevance of NLN and TLN number for TRG. The median number of NLNs and TLNs was 6.89 and 11.45 in the poor tumor regression group (TRG0–1), and 7.37 and 9.90 in the good tumor regression group (TRG2–4), respectively. Although the difference between the poor and good tumor regression groups with respect to NLNs (*t* = –0.465, *P* = 0.694) and TLNs (t = 1.517, *P* = 0.132) was not statistically different, it is interesting to note that, contrary to the decreased number of TLN counts, there were more NLNs in the good tumor regression group compared with the poor tumor regression group.

## DISCUSSION

Metastasis to regional LNs is one of the most important prognostic factors of colorectal cancer [19, 20], and LN assessment is fundamental in nearly all pathological staging systems for colorectal cancer. The number of TLNs evaluated has been associated with RCSS in patients with stage III colorectal cancer; the more nodes identified, the better the long-term survival [20–22]. However, it has been suggested that the TLNs harvested may significantly decrease after preop-RT [3–6] and there are controversial results regarding the effect of TLN retrieval on patient CSS. Our data also shows that TLN counts decrease following preop-RT, but this was not a prognostic factor using the current cutoff standard (≥12 LNs retrieved). The number of NLNs has been confirmed as an independent prognosis factor in colon [14], gastric [15], esophageal [16], and cervical cancer. In our large population based study, we found that the number of NLNs was an independent prognosis factor for ypIIIB and ypIIIC rectal cancer, and even in ypN1 and ypN2 patients, increased numbers of NLNs were associated with improved RCSS. The number of NLNs had a weak or negligible correlation with PLN counts, which means that it was a prognostic factor independent of current metastatic LNs count-based staging. Interestingly, and contrary to the decreased number of TLNs in the good tumor regression group compared with the poor regression group, the number of NLNs was slightly greater in the good tumor regression group than the poor regression group, which suggests that NLN count is a prognostic factor independent of TRG score. Despite this correlation, the mechanism underlying the relationship between the number of NLNs and survival has not been determined, although several hypotheses have been proposed.

The first hypothesis involves stage-migration. A previous study using the SEER data showed that there was continuous worsening survival from stage IIIA to IIIC in rectal cancer patients [23]. Our study indicated that there was a significant correlation between the number of TLNs and NLNs; the more LNs examined, the more likely that it reflects the true stage, and lower nodal counts may increase the risk of understaging. It might also be the case that, after a certain cutoff limit, a further increase in the number of NLNs examined will not have any influence on the accuracy of staging and survival; therefore, we identified 10 as the optimal cutoff value. For stage IIIA patients, which includes T1N1–2a and T2N1 patients, in a large population based study of rectal cancer including 9,566 T1 stage and 10,496 T2 stage patients, only 24 (0.25%) of T1 stage patients had more than seven LNs metastasis (N2b stage) and 422 (4.02%) T2 stage patients had more than four LNs metastasis (N2 stage) [23]. Therefore, the chance of understaging is very low and the value of NLN counts for prognosis is not apparent.

The second hypothesis revolves around the notion that the surgeon is a technician. For instance, preop-RT will cause an immune response and fibrosis in LNs [24], and it may also decrease the size of non-metastatic LNs by 1–2 mm [25–27], thereby decreasing the likelihood of their detection in surgical specimens. It is possible that the patients who had more LNs identified in their specimens experienced more complete excision of their tumors and draining nodes. Improved surgical techniques may also be the result of improved intraoperative staging, to exclude stage IV patients [20] and to reduce the chances of iatrogenic spread of cancer cells. As such, there is less likelihood of leaving tumor cells behind, thus positively affecting survival. Hospital and surgical procedure volume have been identified as predictors of outcome following rectal cancer resection [28, 29]. Thus, a greater number of recovered NLNs may be an indicator of quality of surgical care or pathology. By increasing NLN counts, the chance of micrometastasis remaining within NLNs, which is a proven prognostic factor [30], may decrease.

The third hypothesis regards the function of LNs. The benefit associated with a higher number of NLNs may simply reflect a host lymphocytic reaction to the tumor, which is associated with LN count [31], and lymphocytic reaction to tumor cells has been associated with longer survival in colorectal cancer [32, 33]. Recent studies have further shown that LNs are smaller in patients dying from tumor recurrence, and the number and size of recovered LNs is related to patient histologic antitumor immune response and tumor growth pattern [34]. Moreover, greater lymphocytic reaction has been associated with a high proportion of microsatellite instability [35, 36], which, in turn, is associated with better RCSS [37]. Patient data from FDSCC also showed that even though the number of TLNs decreased, the number of NLNs did not, which maybe an indicator of host immune response to tumor cells, thereby imparting an independent effect on survival.

Although the present study is a large population-based study, it has several potential limitations. First, the SEER database does not include information regarding the administration of chemotherapy and the quality of surgical care or pathological technique, and all of these factors may affect LN harvest. Second, the SEER database is a public cancer registry data, so we cannot further study the mechanisms underlying relationships between the number of NLNs and RCSS. Third, all patients included in this study had received preop-RT, thus our findings cannot be generalized to those that did not receive preop-RT.

In conclusion, our study shows that the number of NLNs was an independent prognostic factor for ypIIIB and ypIIIC rectal cancer patients, and, together with the number of PLNs, it provides more accurate prognostic information than the number of PLNs alone. For survival benefit, we suggest at least 10 NLNs should be retrieved from ypIII rectal cancer patients.

## MATERIALS AND METHODS

### Patient selection in the SEER database

The SEER Cancer Statistics Review (http://seer.cancer.gov/data/citation.html), a report on the most recent cancer incidence, mortality, survival, prevalence, and lifetime risk statistics, is published annually by the Data Analysis and Interpretation Branch of the National Cancer Institute, MD, USA. The current SEER database consists of 17 population-based cancer registries that represent approximately 28% of the population in the US. It contains no identifiers and is publicly available for studies of cancer-based epidemiology and LNs staging of colorectal [14, 23, 38], gastric [39], esophageal [40], and other cancers.

Using the SEER-stat software (SEER*Stat 8.1.2), we searched for patients diagnosed between 1998 and 2005 with single primary rectal cancer (C20.9-Rectum, NOS) and a known treatment sequence consisting of “radiation prior to surgery” or “radiation before and after surgery”. Histological type were limited to adenocarcinoma (8150/3, 8210/3, 8261/3, 8263/3), mucinous adenocarcinoma (8480/3), and signet ring cell carcinoma (8490/3). Age was limited to between 18 and 80 years old. Patients diagnosed after 2006 were excluded to ensure adequate follow-up time. Other exclusion criteria were as follows: ypN0 stage, synchronous distance metastases, and patients who died within 30 days after surgery.

### Patient selection in the FDSCC set

The FDSCC rectal cancer dataset was built prospectively and recorded the rectal cancer patients treated at FDSCC, Shanghai, China since January, 2006. To validate the findings from the SEER database and to clarify relevant issues, we used data from the FDSCC treated with NCRT between January 2006 and December 2012. All patients had histologically confirmed, locally advanced rectal cancer located within 10 cm of the anal verge. Other patient inclusion criteria were: rectal cancer as a single primary tumor, completed NCRT and received radical surgery, and pathologic yp-stage III. Patients that received local resection were excluded from this study. All patients received intensity-modulated radiation therapy to the pelvis of 45–50 Gy and a concomitant boost of 5 Gy to the primary tumor in 25 fractions, concurrent with capecitabine or 5-FU based chemotherapy. Radical surgery was scheduled 6–8 weeks after NCRT. Regression of the primary tumor was semi-quantitatively determined by the amount of viable tumor versus the amount of fibrosis, ranging from no evidence of any treatment effect to a complete response with no viable tumor identified, as described by Dworak et al [41]. The following were characteristics of each grade: grade 0, no regression; grade 1, minor regression (dominant tumor mass with obvious fibrosis in 25% or less of the tumor mass); grade 2, moderate regression (dominant tumor mass with obvious fibrosis in 26% to 50% of the tumor mass); grade 3, good regression (dominant fibrosis outgrowing the tumor mass; i.e., more than 50% tumor regression); and grade 4, total regression (no viable tumor cells, only fibrotic mass) [9].

The research protocol was reviewed and approved by the Ethical Committee and Institutional Review Board of the FDSCC. All patients in FDSCC provided written informed consent.

### Statistical Analysis

Age, sex, race, extension of primary tumor invasion, TLNs, NLNs, PLNs, histological grade, survival time, and CSS were extracted from the SEER database and FDSCC set. All cases were restaged according to the criteria described in the American Joint Committee on Cancer (AJCC) Cancer Staging Manual (7th edition, 2010). Patients within each AJCC substage were dichotomized based on the number of NLNs. The rate of rectal cancer death was compared between the two groups for each substage using the Kaplan–Meier method. Multivariable Cox regression models were built for analysis of risk factors for survival outcomes. The primary endpoint of this study was RCSS, which was calculated from the date of diagnosis to the date of cancer specific death. Deaths attributed to the rectal cancer were treated as events and deaths from other causes were treated as censored observations. All of statistical analyses were performed using the statistical software package SPSS for Windows, version 17 (IBM Corp, Armonk, NY, USA). Statistical significance was set at two-sided *P* < 0.05.
